# Evaluation of a systematic approach to weaning of tracheotomized neurological patients: an early interrupted randomized controlled trial

**DOI:** 10.1186/s13613-015-0098-0

**Published:** 2015-12-23

**Authors:** Rosanna Vaschetto, Pamela Frigerio, Maurizio Sommariva, Arianna Boggero, Valentina Rondi, Francesca Grossi, Silvio Cavuto, Stefano Nava, Francesco Della Corte, Paolo Navalesi

**Affiliations:** Anesthesia and Intensive Care Medicine, Maggiore della Carità Hospital, Corso Mazzini 18, 28100 Novara, Italy; Dipartimento di Neuroscienze, Azienda Ospedaliera Niguarda Ca’ Granda, Piazza Dell’Ospedale Maggiore 3, 20162 Milano, Italy; Dipartimento di Medicina Traslazionale, Università del Piemonte Orientale “Amedeo Avogadro”, Alessandria-Novara-Vercelli, via Solaroli 17, 28100 Novara, Italy; Department of Infrastructure Research and Statistics, IRCCS-Arcispedale Santa Maria Nuova, Viale Umberto I 50, 42123 Reggio Emilia, Italy; Respiratory and Critical Care, Department of Specialistic, Diagnostic and Experimental Medicine (DIMES), Alma Mater Studiorum, Sant’Orsola Malpighi Hospital, University of Bologna, Via Zamboni 33, 40126 Bologna, Italy; Anesthesia and Intensive Care Medicine, Sant’Andrea Hospital, C.so M. Abbiate 21, 13100 Vercelli, Italy; CRRF Mons. L. Novarese, Moncrivello, Localita’ Trompone, 13040 Vercelli, Italy

**Keywords:** Weaning, Tracheotomy, Brain-injured patients

## Abstract

**Background:**

While a systematic approach to weaning reduces the rate of extubation failure in intubated brain-injured patients, no data are available on the weaning outcome of these patients after tracheotomy. We aimed to assess whether a systematic approach to disconnect tracheotomized neurological and neurosurgical patients off the ventilator (intervention) is superior to the sole physician’s judgment (control). Based on previous work in intubated patients, we hypothesized a reduction of the rate of failure within 48 h from 15 to 5 %. Secondary endpoints were duration of mechanical ventilation, ICU length of stay and mortality.

**Methods:**

We designed a single center randomized controlled study. Since no data are available on tracheotomized patients, we based our a priori power analysis on results derived from intubated patients and calculated an overall sample size of 280 patients.

**Results:**

After inclusion of 168 consecutive patients, the trial was interrupted because the attending physicians judged the observed rate of reconnection to be much greater than expected. The overall rate of failure was 29 %, confirming the physicians’ judgment. Twenty-one patients (24 %) in the intervention group and 27 (33 %) controls were reconnected to the ventilator within 48 h (p = 0.222). The main reasons for failure were respiratory distress (80 and 88 % in the treatment and control group, respectively), hemodynamic impairment (15 and 4 % in the treatment and control group, respectively), neurological deterioration (4 % in the control group only). The duration of mechanical ventilation was of 412 ± 202 h and 402 ± 189 h, in the control and intervention group, respectively. ICU length of stay was on average of 23 days for both groups. ICU mortality was 6 % in the control and 2 % in the intervention group without significant differences.

**Conclusion:**

We found no difference between the two groups under evaluation, with a rate of failure much higher than expected. Consequent to the early interruption, our study results to be underpowered. Based on the results of the present study, a further trial should overall enroll 790 patients.

Trial registration: ACTRN12612000372886

## Background

Both weaning failure [1] and extubation failure [2] determine prolonged mechanical ventilation, which increases morbidity [3–6] and mortality [6, 7] of intensive care unit (ICU) patients.

A systematic approach to weaning and extubation incorporating assessment of neurological function and ability to cough and clear secretions [8] was proved to decrease the rate of extubation failure in intubated patients with neurological disorders. A considerable rate of these patients, however, may undergo tracheotomy [9], which eliminates the detrimental consequences of extubation failure and re-intubation, but does not necessarily affect the process of withdrawing mechanical ventilation.

Because no study has so far evaluated the efficacy of a systematic approach to wean the tracheotomized patients off the ventilator, we designed this randomized controlled trial to ascertain whether a systematic approach based on daily screening of meaningful physiologic and clinical variables followed by a spontaneous breathing trial (SBT), is superior to direct disconnection off the ventilator based on the sole physician’s judgment in reducing the rate of reconnection to the ventilator within 48 h (primary endpoint). Secondary endpoints were duration of mechanical ventilation, ICU length of stay (LOS) and ICU mortality.

## Methods

### Patients and setting

The study was performed in the ICU of the University Hospital “Maggiore della Carità” (Novara, Italy) between January 2011 and December 2013, according to the principles outlined in the Declaration of Helsinki. The ICU is a closed fourteen-beds unit, attended around the clock by physicians all certified and trained in Anesthesiology and Critical Care. The protocol was approved by the local Ethics Committee i.e., Comitato Etico Interaziendale A.O.U. “Maggiore della Carità”, ASL BI, ASL NO, ASL VC, ASL VCO (Corso Mazzini n. 18, 28100 Novara; Protocol 145/CE; Study n. CE 21/11), and written informed consent to participate in the study and to publish results was obtained for all the patients according to the Italian regulations. In our routine care, irrespective of the cause of intubation and mechanical ventilation, tracheotomy is considered after 7 days of invasive mechanical ventilation for the patients for whom extubation is considered unlikely to occur in the following 3–5 days. If there is uncertainty or disagreement at this regard, the patient is re-evaluated after 2–3 additional days. Tracheotomy is performed, anyway, within 14 days after initiation of invasive mechanical ventilation in all patients. At the time of the study, there were no formal protocols for either weaning or disconnection from the ventilator. The weaning strategy for tracheotomized patients was based on the application of pressure support ventilation with progressive reduction of positive end-expiratory pressure (PEEP) and support level; the decision of disconnecting the patient was left to the discretion of the attending physician in charge.

Patients who met the following inclusion criteria were considered eligible: 1) diagnosis at ICU admission of neurosurgical and neurological disease, 2) presence of a tracheotomy performed in our ICU; 3) age >18 years, 4) no need for continuous intravenous sedative infusion and/or controlled mechanical ventilation, 5) ability to trigger the ventilator, 6) no scheduled surgery in the following 72 h. The only exclusion criteria were the preexisting decision to limit life support and the inclusion in other research protocols.

After enrolment, patients were assigned to the intervention or control group following a previously generated random sequence held by an investigator not involved in the study enrolment, who indicated in sealed, opaque numbered envelops the group of assignment. The trial was registered at the Australian New Zealand Clinical Trials Registry (ACTRN12612000372886).

### Study protocol and measurements

Routine care was no different between the two groups. Active humidification was used for all patients both during mechanical ventilation and during spontaneous breathing, while tracheal suctioning and mechanically assisted cough devices were applied when needed. All patients randomized to treatment were screened every morning to assess readiness for SBT by the attending physicians. The criteria for readiness were 1) normal sodium blood values, 2) core temperature <38.5 °C during the previous 8 h, 3) pH ≥ 7.35, 4) a ratio of partial pressure arterial oxygen and fraction of inspired oxygen (PaO_2_/FiO_2_) ≥200 mm Hg with a PEEP ≤5 cm H_2_O, 5) FiO_2_ ≤0.4, 6) heart rate ≤125 beat/min, 7) systolic blood pressure (SBP) ≥90 mmHg without epinephrine or norepinephrine infusion and with dopamine infusion ≤5 µg/kg/min.

The patients who passed the screening underwent a 30 min SBT through the circuit of a flow-triggered ventilator, set to deliver 2 cm H_2_O of continuous positive airway pressure, with a FiO_2_ of 0.4. The trial was interrupted and mechanical ventilation resumed whenever one of the following occurred: 1) respiratory rate >35/min, 2) signs of evident respiratory distress (diaphoresis, accessory muscles recruitment, thoraco-abdominal paradox), 3) peripheral oxygen saturation (SpO_2_) <90 %, 4) SBP <90 mmHg or >180 mmHg, 5) heart rate >140/min.

At the end of the trial, the patients who had a respiratory rate/tidal volume ratio (RR/VT) ≤105 and PaO_2_/FiO_2_ ≥200 mm Hg with a pH ≥ 7.35 were immediately disconnected from the ventilator. Conversely, if the RR/VT exceeded 105 or the arterial blood gas analysis criteria were not met, mechanical ventilation was resumed. Patients randomized to the control group, as routine use in the ICU, were evaluated every day by the attending physicians who discontinued mechanical ventilation based on clinical judgment; all the information collected for the intervention group were also available.

Irrespective of the group of randomization, patients were considered successfully weaned off if they did not meet criteria for resuming mechanical ventilation within 48 h. Criteria for ventilation resumption were 1) emergency, such as respiratory or cardiac arrest, and gasping for air, 2) neurologic deterioration [a decrease of more than 2 points of Glasgow Coma Scale (GCS)], 3) hemodynamic instability (i.e., need for continuous infusion of epinephrine, norepinephrine or vasopressin, or dopamine  >5 µg/kg/min to maintain SBP >90 mmHg), despite adequate filling, 4) respiratory distress, as assessed by the combination of SpO2 <90 %, respiratory rate >35/min, and visible accessory muscle recruitment or thoraco-abdominal paradox, despite administration of oxygen.

### Statistical analysis

Lacking data on tracheotomized patients, we used the sample size of 280 patients (140 per group to detect a decrease in failure i.e., reconnection to the ventilator within 48 h, from 15 to 5 % with 80 % power at the 5 % two-sided level of significance) previously adopted for intubated patients [8]. Normally distributed variables were compared by two-tailed Student’s *t* test, non-parametric variables were compared by Mann–Whitney test, whereas proportions and rates by Fisher’s exact test, as indicated. Data were analyzed according to intention-to-treat analysis.

## Results

**Fig. 1:**
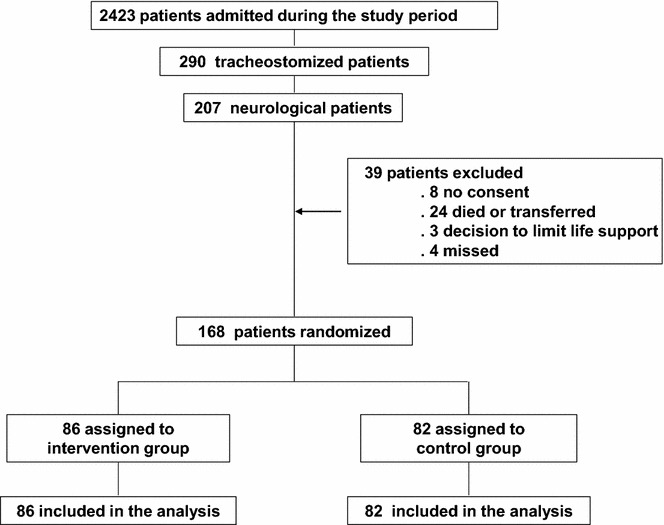
Flow of patients through the trial

As depicted in the trial profile in Fig. 1, 290 of 2423 patients admitted during the study period underwent tracheotomy. Of these, 207 were neurological or neurosurgical patients, 168 of who were included in the study protocol and randomized to either the intervention (86 patients) or control group (82 patients). As shown in Table 1, patients’ characteristics at ICU admission and reasons for admission were similar between groups. GCS at the time of disconnection off the ventilator was identical (8 ± 3). PaO_2_/FiO_2_ was 310 ± 66 mmHg and 306 ± 63 mmHg in the intervention and control group, respectively (*p* = 0.74). There was one protocol violation in the treatment group (patient disconnected despite PaO_2_/FiO_2_ <200 mmHg).

**Table 1:** Patients characteristics at enrolment and reason for ICU admission

	Intervention group (n = 86)	Control group (n = 82)
Patients characteristics		
Age, year, mean (SD)	54 (17)	54 (19)
Male/female, n	50/36	47/35
SAPS II score, mean (SD)	45 (13)	44 (16)
GCS at ICU admission, mean (SD)	6 (3)	7 (4)
GCS at disconnection, mean (SD)	8 (3)	8 (3)
PaO_2_/FiO_2_ at disconnection, mean (SD)	310 (66)	306 (63)
Reason for ICU admission		
Subarachnoid/intracerebral hemorrage, n (%)	45 (52)	48 (59)
Head trauma, n (%)	27 (31)	23 (28)
Cerebral tumor, n (%)	4 (5)	1 (1)
Other, n (%)	10 (12)	10 (12)

*GCS* Glasgow Coma Scale, *ICU* intensive care unit, *PaO*_*2*_ */FiO*_*2*_ ratio of partial pressure arterial oxygen and fraction of inspired oxygen, *SAPS* simplified acute physiologic score, *SD* standard deviation

After enrollment of 168 patients, corresponding to 60 % of the total planned recruitment, the attending ICU physicians, who were aware of the expected rates of failure, informed the researchers that in their opinion the actual rate of failure exceeded by far that expected. For this reason the investigators decided to undertake an interim analysis; the recruitment of patients was then interrupted and the data analyzed. The overall rate of failure was 29 %, confirming the physicians’ judgment. As shown in Table 2, 21 patients (24 %) in the intervention group and 27 (33 %) controls were reconnected to the ventilator within 48 h (*p* = 0.222). The main reasons for failure were respiratory distress (80 and 88 % in the treatment and control group, respectively), hemodynamic impairment (15 and 4 % in the treatment and control group, respectively), neurological deterioration (4 % in the control group only); in one patient in the treatment group the reason for reconnection was not recorded. As also shown in Table 2, duration of mechanical ventilation, ICU-LOS and mortality were not significantly different between the two groups. Additionally, irrespective of the group of randomization, there were no significant differences between the patients who failed and those who succeeded with respect to duration of mechanical ventilation (18 ± 7 vs. 16 ± 8 days for failure and success, respectively, *p* = 0.197), ICU-LOS (25 ± 10 vs. 22 ± 9 days for failure and success, respectively, *p* = 0.065), and ICU mortality (6 vs. 3 % for failure and success, respectively, *p* = 0.392).

**Table 2:** Comparison of outcomes between study groups

	Intervention group (n = 86)	Control group (n = 82)	*P*
Primary endpoint			
Weaning failure at 48 h, n (%)	21 (24)	27 (33)	0.222
Secondary endpoints			
Duration of MV, hours, mean (SD)	402 (189)	412 (202)	0.972
ICU length of stay, days, mean (SD)	23 (9)	23 (10)	0.913
ICU mortality, n. (%)	2 (2)	5 (6)	0.228

*ICU* intensive care unit, *MV* mechanical ventilation, *SD* standard deviation

After study interruption, we re-calculated the sample size based on the actual rates of failure and found that 790 patients would be overall necessary to properly set a future study, which makes this clinical trial unfeasible in a single center.

## Discussion

As our trial aborted, it does not supply valuable information on the predetermined endpoints. Paradoxically, however, our study provides novel information of potential interest. While the rate of failure in analogous populations of intubated brain-injured patients does not exceed 16 % [8, 10], the rate of reconnection to the ventilator in the present study is 29 %. To our knowledge, this information has never been reported before. Respiratory distress is the most common reason for resuming mechanical ventilation in the majority (85 %) of our tracheotomized patients. In an analogous population of intubated patients, respiratory distress also resulted to be the most common determinant of failure, although with a much lower rate (41 %) [8].

While re-intubation consequent to weaning and extubation failure [2, 8, 11–13] or unplanned extubation [14, 15] is associated with longer ICU-LOS and increased risk of death both in neurological patients [8, 11] and in a general ICU population [2, 11–13, 15], in the present study, we observed no significant difference in any of these clinical outcome variables between the patients who were successfully weaned off the ventilator and those who failed. These findings suggest that failing disconnection off the ventilator in tracheotomized patients has not the same deleterious effects observed in intubated patients after extubation failure, confirming that in these latter patients the worsened outcome is a direct and specific effect of extubation failure and re-intubation [15]. Nonetheless, as all the patients who failed the disconnection off the ventilator had passed a protocolized SBT or were judged ready to sustain spontaneous breathing by the ICU physician, we cannot exclude different results for earlier disconnections owing to the easiness to reconnect the patient.

The actual rate of failure in disconnecting patients from the ventilator encountered in the present trial may represent valuable data for designing a future adequately powered multicenter randomized controlled trial. However, the lack of differences in outcome between the patients who failed and those who succeeded disconnection, as opposed to the findings of previous studies on intubated patients [8, 11], makes disconnection failure in brain-injured tracheotomized clinically unimportant and limit by far the interest for possible strategies aimed at minimizing it. Therefore, rather than assessing the impact of a protocolized strategy on the outcome of disconnection, the aim of a future study would better be confirming that failure is not associated with a worse prognosis.

Beside the insufficient sample size consequent to early study interruption, our study has other limitations. First, because the study was conducted in a single center, our findings might be not applicable to other situations. Second, because the study could not be blinded, we cannot exclude some carry-over effect occurred. Third, our results could be influenced by the criteria adopted to assess readiness and to determine SBT success or failure, and we cannot exclude that the results may have been different by adopting additional criteria more adapted to the specificities of our population.

## Conclusions

In brain-injured patients undergoing mechanical ventilation through a tracheotomy, we found the rate of reconnection to the ventilator much higher than expected, which led us to early interruption of the trial. Although underpowered, our study suggests that a systematic approach to weaning through daily screening using a written flow chart followed by a SBT does not significantly reduce the rate of reconnection to the ventilator, as opposed to direct disconnection based on physician’s judgment only. Duration of mechanical ventilation, ICU-LOS and ICU mortality showed no significant difference either between intervention group or controls and between the patients who failed and those who succeeded, regardless of the assignment group.

## Abbreviations

GCS: Glasgow Coma Scale
ICU: intensive care unit
LOS: length of stay
PaO_2_/FiO_2_: ratio of partial pressure arterial oxygen and fraction of inspired oxygen
PEEP: positive end-expiratory pressure
RR/VT: respiratory rate/tidal volume ratio
SAPS: simplified acute physiologic score
SBP: systolic blood pressure
SBT: spontaneous breathing trial
SD: standard deviation
SpO_2_: peripheral oxygen saturation
